# Supplementary figures and images for: Application of ARIMA, and hybrid ARIMA Models in predicting and forecasting tuberculosis incidences among children in Homa Bay and Turkana Counties, Kenya
Source: PLOS Digit Health. 2023 Feb 1;2(2):e0000084. doi: 10.1371/journal.pdig.0000084 (PMC9931286; doi:10.1371/journal.pdig.0000084)

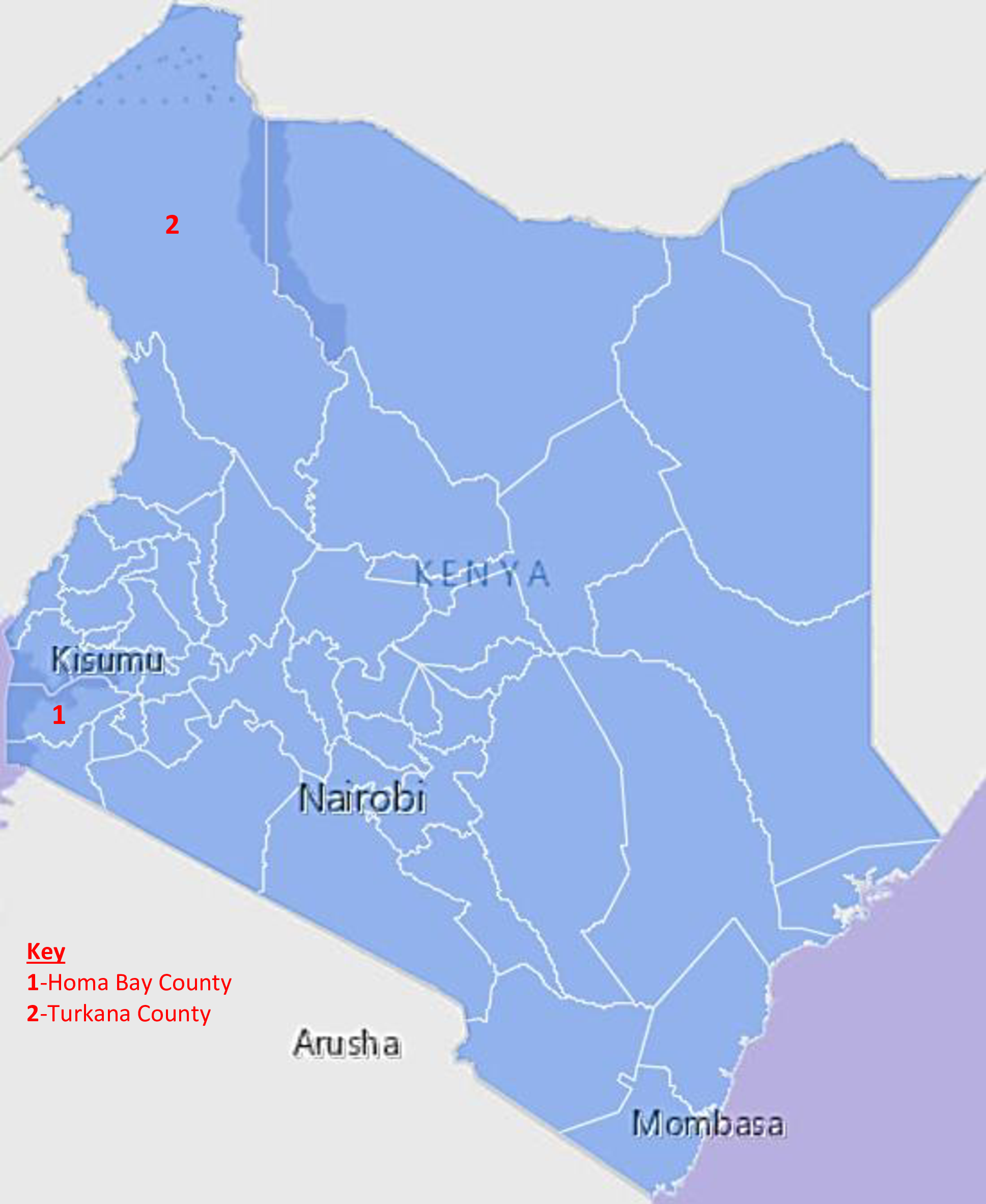

Supplement: S1 Fig — (https://kenya.africageoportal.com/datasets/d2f2df2a08ef42e88cb6bdc00e41dcc9_0/explore?location=0.361948%2C41.711735%2C6.00) [31]. (TIF) [file pdig.0000084.s001.tif]

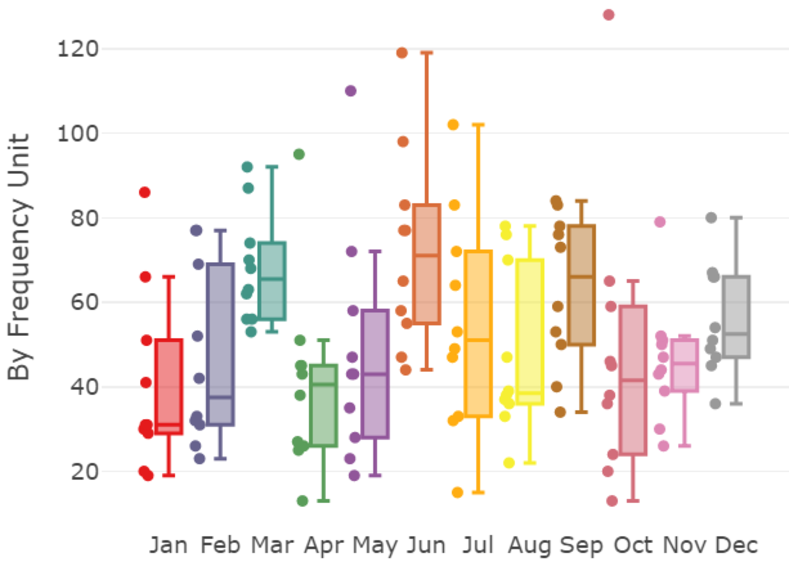

Supplement: S2 Fig — (TIF) [file pdig.0000084.s002.tif]

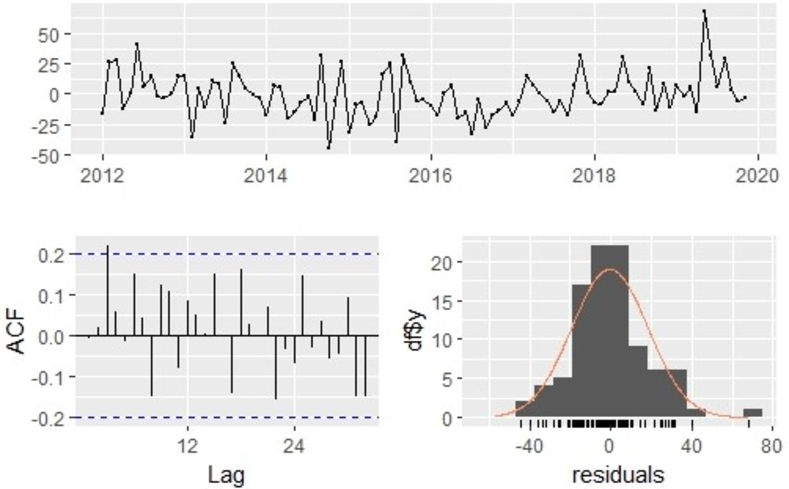

Supplement: S3 Fig — (TIF) [file pdig.0000084.s003.tif]
