# Supplementary material for: Application of ARIMA, and hybrid ARIMA Models in predicting and forecasting tuberculosis incidences among children in Homa Bay and Turkana Counties, Kenya
Source: PLOS Digit Health. 2023 Feb 1;2(2):e0000084. doi: 10.1371/journal.pdig.0000084 (PMC9931286; doi:10.1371/journal.pdig.0000084)
Supplement: Attachment — Submitted filename: PDIG-D-22-00198.docx [file pdig.0000084.s005.docx]

The authors developed ARIMA, Hybrid ARIMA and ANN models to predict and forecast the incidence of Tuberculosis among under 15 children. The topic is interesting, primarily focusing on paediatric Tuberculosis.

1. Since the number of data points is significantly less (120) and from the methodology, the authors used an 80/20 split for training and testing, it is unclear whether the ARIMA or hybrid ARIMA orders are determined after splitting the training and test set or using the whole dataset. If authors used the entire dataset for determining the order, they could introduce the bias in the test set. What are the measures taken to mitigate the overfitting issues?
2. It is unclear whether the authors used any cross-validation techniques in this work. Many applicable packages are available in R for utilizing the cross-validation for better performance in predicting and forecasting, especially when the data points are less.
3. Model (ARIMA (0,0,1,1,0,1,12)), Model (NNAR (1,1,2) [12]), and Hybrid ARIMA-ANN shows the test errors are higher than training errors. Even though it is common in models, the large variation indicates the overfitting of the model. Can the authors explain the measures taken to reduce the test errors?
