# Supplementary material for: Application of ARIMA, and hybrid ARIMA Models in predicting and forecasting tuberculosis incidences among children in Homa Bay and Turkana Counties, Kenya
Source: PLOS Digit Health. 2023 Feb 1;2(2):e0000084. doi: 10.1371/journal.pdig.0000084 (PMC9931286; doi:10.1371/journal.pdig.0000084)
Supplement: Attachment — Submitted filename: Response to Reviewers.docx [file pdig.0000084.s006.docx]

Thomas Schmidt, PhD

Academic Editor

PLOS Digital Health

November 3, 2022

Dear Dr. Thomas Schmidt,

Thank you for the opportunity granted to revise this manuscript: PGPH-D-22-00198 “**Application of ARIMA, hybrid ARIMA and Artificial Neural Network models in predicting and forecasting tuberculosis incidences among children in Homa Bay and Turkana Counties, Kenya**”. We would like to thank the reviewers for their comments. We have revised our manuscript accordingly. Please find below point-by-point responses in ***bold italics*** to the reviewers’ comments.

**Reviewers’ Comments**

**Additional Editor Comments**

Foremost regarding the structure and content of the paper. I’m of the opinion that research papers should limit the elaborate use of formulas for fairly generic algorithms. They make sense when adjustments or customizations have been utilized. So please consider if the many equations listed in Materials and methods section are truly necessary, or can be found in textbooks. Likewise, I recommend a reconsideration of the value of listing metrics for models during both training and testing. These metrics are useful when doing an overall evaluation of each model’s performance, but consider if they are relevant for potential readers.

**Thank you for these comments**

**We reviewed the equations presented and adjusted accordingly. We ensured to reduce the number of equations to those that are essential and referred to better direct the reader**

I find your paper to be well written but would like to have you elaborate the discussion a bit further on the applicability of your approach.

**We appreciate this comment and suggestion**

**We proceeded to elaborate further in the discussion the implication and applicability of our approach. We have included additional discussion on the correlation between the TB cases as well as the forecasted cases from this study and seasonal patterns.**

Also, to what extent is your dataset affected by COVID-19? Please consider this in your limitations section.

**Thank you for this comment**

**We acknowledge the role that COVID-19 pandemic and the measures that were put in place to limit the spread of the virus. We have taken account of this within the limitations of this study**

Finally, I suggest that you use consistent terminology for your dataset. ‘Data points’ are confusing, please use subjects, patients, or children instead. You use both data points, records etc.

**Thank you for the comment. We have made the corrections where applicable**

**Reviewer #1**

**Specific comments**

This study applied ARIMA, hybrid ARIMA and ANN to predict incidence of TB in Children in two counties in Kenya and demonstrates that hybrid ARIMA has better predictive and forecast accuracy in comparison to ARIMA and ANN models. This is an interesting study and including more details in methods would improve the manuscript further.

***Thank you for the comments***

***We have revised our methods further to take account of the review comments especially in making the methods more robust by implementing the cross-validation technique.***

***Further, we felt the need to include a sub-section on the study setting to make clearer and more elaborated more on the proposed methodology***

**Major comments #1**

The authors haven’t provided adequate justification for this study apart from stating that previous studies have used only ARIMA models in forecasting disease incidence in Kenya. It would be helpful if the authors conducted a more rigorous literature review to identify gaps in literature on how ARIMA and hybrid ARIMA models were applied to forecasting disease incidence in Africa and Kenya. Also, the authors must highlight the gaps in Azeez et al.’s study and the methodological gaps in that study to justify the aims and objectives of this study

***Thank you for this comment.***

***We have carried out further literature review in addition to what we had included in the manuscript earlier.***

***We have included insights from Alfred and Obit (2021) on the role of machine learning in infectious disease surveillance, Langat et al. (2017), Ebhuoma et al. (2018) and Anokye et al. (2018) who use ARIMA based models to model non-infectious diseases, and Suleiman and Sani (2020) and Takele (2020) on more recent utilization of ARIMA models in modelling of COVID-19 infections within the context of infectious diseases.***

***We also reviewed Umunna and Olanrewaju (2020) who utilized ARIMA based models to model HIV cases, and Ade et al. (2016) who utilize ARIMA models to model TB infections in addition to Aryee et al. (2018). In each of these reviews, we have highlighted on the methodology used, the results where possible and possible short-comings of the methods used which was informed partly by the limitations within these literature***

My biggest concern with this study is the selection of ANN models as a comparator. With the limited size of the dataset, these models were always likely to underperform. Adequate justification on why these models have been used for this study and why it is important to compare ANN with ARIMA and hybrid ARIMA would be helpful. Also, multiple other studies have demonstrated that SARIMA

***Thank you for this comment.***

***After a thorough review of the implications of developing ANN model and comparing with the ARIMA and hybrid ARIMA, we decided to drop the ANN model from the list of models considered within the study. We realized that the ANN model performed worse on the test data majorly because of limited learning attributed to the amount of data available. Furthermore, we decided to subject the ANN model development to cross-validation techniques in order to obtain the best accuracy measures but we did not fare any better.***

***However, with availability of enough data, the ANN model could be given future consideration.***

I would strongly encourage the authors to use Jandoc et al.’s study (<https://pubmed.ncbi.nlm.nih.gov/25890805/>) and Schaffer et al.’s article (<https://bmcmedresmethodol.biomedcentral.com/articles/10.1186/s12874-021-01235-8> ) to report the methodological and reporting recommendations

***Thank you for the recommendations on Jandoc et al.***

***We find this particular study interesting and thought provoking especially from the perspective of analysis of equally spaced time series where there is an intervention and with an objective of assessing the impact of an intervention within the scope of drug utilization research. Although our study does not fit within drug utilization research, we feel that interrupted time series analysis can be considered as well in the case of interruptions rather than interventions. In our study, this particular methodology can be applied in assessing the impact of COVID-19 on a previous TB trend and basically determine whether progress would have been sustained in the absence of such an interruption. We feel that we can apply this method and develop an additional manuscript that can assess the potential impact of COVID-19 in TB case identification among children and within a wider scope. We also think this particular methodology can be made more robust through approaches such as simulation as well as validation techniques in order to obtain best accuracies.***

***On Schaffer et al., this study is quite interesting and we were able to pick some pointers that could improve our study; (1) In our study, the series was determined to be stationary, however, this should be considered as weakly stationary with the possibility of accounting for it by setting d=1, (2) Since our study utilized monthly TB data, the possibility of seasonality and the need to account for it is important.***

To test the ARIMA, hybrid ARIMA and ANN, it is not clear if the authors conducted a sensitivity analysis

***Thank for this comment***

***Initially, we did not consider sensitivity analysis. However, upon further consideration, we decided to amend our methodology to allow for cross-validation which would allow the best model to be considered based on accuracy parameters and also allow the establishment of the best forecast horizon that would result in the lowest errors.***

**Minor comments**

In line 204, did the authors mean ARMA (Autoregressive Moving Average) model or ARIMA model? If it is the former, please expand ARMA

***Thank you for this comment***

***This has been corrected, we meant ARIMA***

I would also encourage the authors to include the data as supplementary material

***Thank you for this comment and suggestion***

***We have included the data as supplementary material***

**Reviewer #2:** The authors developed ARIMA, hybrid ARIMA and ANN models to predict and forecast the incidence of Tuberculosis among under 15 children. The topic is interesting, primarily focusing on paediatric Tuberculosis

**Comments #1**

Since the number of data points is significantly less (120) and from the methodology, the authors used an 80/20 split for training and testing, it is unclear whether the ARIMA or hybrid ARIMA orders are determined after splitting the training and test set or using the whole dataset. If authors used the entire dataset for determining the order, they could introduce the bias in the test set. What are the measures taken to mitigate the overfitting issues?

***Thank you for this comment.***

***We recognize the risk of introducing bias within the testing dataset and as such, the ARIMA orders were determined on the training dataset and not the whole set even within the amended methodology where we have used cross-validation technique.***

***In order to address the issue of overfitting, we had initially thought that utilizing the training set for model development and the testing set for model validation would sufficiently suffice. However, based on reviewer comments, we felt that addressing overfitting and/or underfitting issues needed to be addressed using a more robust and novel way. As such, we implemented, based on reviewer recommendation, cross-validation***

**Comments #2**

It is unclear whether the authors used any cross-validation techniques in this work. Many applicable packages are available in R for utilizing the cross-validation for better performance in predicting and forecasting, especially when the data points are less.

***Thank you for this comment.***

***We recognize the importance of having to model using fewer data points and how this can have a significant effect on model accuracy and generalization of the model findings.***

***At the initial phase of developing this manuscript, we utilized the ‘traditional’ method of using training and testing data in model development and validation respectively. We however, recognize the this might not have been adequate especially with the disadvantage of having* *few data points, to properly address model accuracy. As such, within this review, we have implemented rolling window cross validation technique and we noticed that the accuracy of the model was better compared to what we had before.***

**Comments #3**

Model (ARIMA (0,0,1,1,0,1,12)), Model (NNAR (1,1,2) [12]), and Hybrid ARIMA-ANN shows the test errors are higher than training errors. Even though it is common in models, the large variation indicates the overfitting of the model. Can the authors explain the measures taken to reduce the test errors?

***Thank you for this comment.***

***First, based on the review provided, we felt the need to drop the NNAR model from this manuscript owing to the gap in terms of data availability to allow for proper learning of the neural network.***

***Furthermore, as indicated earlier our response, we decided to implement a cross-validation procedure during model development in order to adequately address model accuracy and realized an improvement***

Respectfully,

Stephen Siamba
